# Mixed Training Methods: Effects of Combining Resisted Sprints or Plyometrics with Optimum Power Loads on Sprint and Agility Performance in Professional Soccer Players

**DOI:** 10.3389/fphys.2017.01034

**Published:** 2017-12-12

**Authors:** Irineu Loturco, Ronaldo Kobal, Katia Kitamura, Cesar C. Cal Abad, Bruno Faust, Lucas Almeida, Lucas A. Pereira

**Affiliations:** ^1^Nucleus of High Performance in Sport (NAR), São Paulo, Brazil; ^2^Grêmio Esportivo Osasco Football Club (GEO), São Paulo, Brazil

**Keywords:** team-sports, football, speed ability, vertical jumps, optimal loads

## Abstract

The aim of this study was to compare the effects of two different mixed training programs (optimum power load [OPL] + resisted sprints [RS] and OPL + vertical/horizontal plyometrics [PL]) on neuromuscular performance of elite soccer players during a short-term training preseason. Eighteen male professional soccer players took part in this study. The athletes were pair-matched in two training groups: OPL + RS and OPL + PL. Unloaded and resisted sprinting speeds at 5-, 10-, 20-, and 30-m, change of direction (COD) speed, and performance in the squat jump (SJ), countermovement jump (CMJ), and horizontal jump (HJ) were assessed pre- and post- a 5-week training period. Magnitude based inference with the effect sizes were used for data analysis. A *possible* increase in the SJ and CMJ heights and a *likely* increase in the HJ distance were observed in the OPL + PL group. Meaningful improvements were observed in the COD speed test for both training groups comparing pre- and post-measures. In both unloaded and resisted sprints, meaningful decreases were observed in the sprinting times for all distances tested. This study shows that a mixed training approach which comprises exercises and workloads able to produce positive adaptations in different phases of sprinting can be a very effective strategy in professional soccer players. Moreover, the possibility of combining optimum power loads with resisted sprints and plyometrics emerges as a novel and suitable option for coaches and sport scientists, due to the applicability and efficiency of this strength-power training approach.

## Introduction

Mixed training methods are widely recognized as efficient alternatives to improve neuromuscular performance during complex abilities such as maximal sprints and jumps (Newton and Kraemer, 1994; Saez de Villarreal et al., 2013). Therefore, coaches and sport scientists have been making significant efforts to identify the best combinations between different modes of strength-power exercises and training strategies (Newton and Kraemer, 1994; Saez de Villarreal et al., 2013; Loturco et al., 2015e, 2016b,c). Recent studies have shown that the concomitant use of traditional lifts (e.g., half squat) and sport-specific movements (e.g., loaded or unloaded jumps) may be highly effective to increase speed and power abilities in athletes from different disciplines and with distinct training backgrounds (Newton and Kraemer, 1994; Maio Alves et al., 2010; Saez de Villarreal et al., 2013). In this context, it seems that the exercise selection plays a key role in determining the magnitude and direction of the expected adaptations (Young et al., 2001). As such, the implementation of functional exercises which better mimic (or at least reproduce) the mechanical characteristics of dynamic sport tasks seems to be an essential and critical issue in elite sports (Young, 2006).

For example, the ability to orient the resultant ground reaction force vector with a forward orientation over the acceleration has been considered as a determinant factor of maximal sprint performance (Morin et al., 2012). From these data, it is plausible that the chronic use of horizontally oriented exercises (e.g., horizontal jumps and resisted sprints) during the training sessions would optimize the positive responses in both speed and acceleration capacities. Indeed, a program exclusively composed of horizontal plyometrics could significantly increase the short-distance acceleration ability (i.e., up to 10 m) in elite young soccer players (Loturco et al., 2015e). Conversely, the “vertical-jump group” reported superior increments in speed at longer distances (i.e., from 10 to 20 m) (Loturco et al., 2015e), which is consistent with the increased and substantial importance of vertical ground reaction forces during the transition from lower to higher velocities (Nilsson and Thorstensson, 1989; Weyand et al., 2000; Markovic et al., 2007). Likewise, resisted sprint training with different workloads (i.e., ranging from 5 to 20% BM) has already been shown to be effective in improving the maximal speed performance of physically trained subjects (in distinct running phases) over a 40 m distance (Bachero-Mena and Gonzalez-Badillo, 2014).

More recently, some authors have suggested the use of the optimum power loads (OPL) (i.e., workloads able to maximize power output) as an alternative way to enhance strength and speed qualities in elite team sport athletes (Loturco et al., 2016a,b,c). Essentially, this recommendation is based on two underlying reasons: (1) the well-established effects of this range of loads (capable of producing significant enhancements at both ends of the force-velocity curve) on athletic capacities (Wilson et al., 1993), and (2) the functional characteristics of this training strategy (which can be controlled in real-time, using the velocity-based training approach) (Bryan et al., 2015; Loturco et al., 2015b). More importantly, it has been reported that this method may meaningfully reduce the concurrent effects between power and endurance adaptations, commonly experienced by professional soccer players during their preseason practices (Loturco et al., 2015d). Despite these positive and encouraging premises, there is a lack of applied research investigating the potential effects of combining OPL with speed-specific exercises such as resisted sprints and plyometric drills (i.e., unloaded vertical and horizontal jumps) in top-level athletes. Considering the multifactorial nature of sprinting speed (Cronin and Hansen, 2005; Markovic et al., 2007; Loturco et al., 2017a), it would be reasonable to assume that more mixed and complex strength-power training approaches would lead to greater and more consistent improvements in this neuromechanical capacity.

Thus, the purpose of this study was to compare the effects of two different mixed training programs (OPL + resisted sprints and OPL + vertical and horizontal plyometrics) on neuromuscular performance of elite soccer players during a short training preseason (i.e., 5 weeks). Owing to the recognized efficacy of these respective exercise strategies (Wilson et al., 1993; Bachero-Mena and Gonzalez-Badillo, 2014; Loturco et al., 2015e), we expected that both training modes would ensue meaningful gains in both speed and power-related capacities.

## Materials and methods

### Participants

Twenty-two male professional soccer players from the same club took part in this study. Players were pair-matched into two training groups according to their baseline performance in the 30-m sprint test as follows: optimum power load + resisted sprint group (OPL + RS; *n* = 11; age: 21.7 ± 2.4 years, height: 176.9 ± 9.0 cm, body-mass [BM]: 73.5 ± 6.2 kg) and optimum power load + vertical and horizontal plyometrics (OPL + PL; *n* = 11; age: 22.2 ± 2.4 years, height: 179.0 ± 5.0 cm, body-mass [BM]: 75.5 ± 11.5 kg). Group allocation was performed by tossing a coin. Four players from the OPL + RS were excluded from the sample due to injuries unrelated to the proposed training/testing or transference to another team. Therefore, 18 players completed the study (*n* = 7 and *n* = 11 for OPL + RS and OPL + PL, respectively). The study protocol took place prior to the first division São Paulo State Soccer Championship, during the preseason training period. The study was approved by the Anhanguera-Bandeirante University Ethics Committee and the participants signed an informed consent form prior to research commencement.

### Experimental design

**Figure 1:**
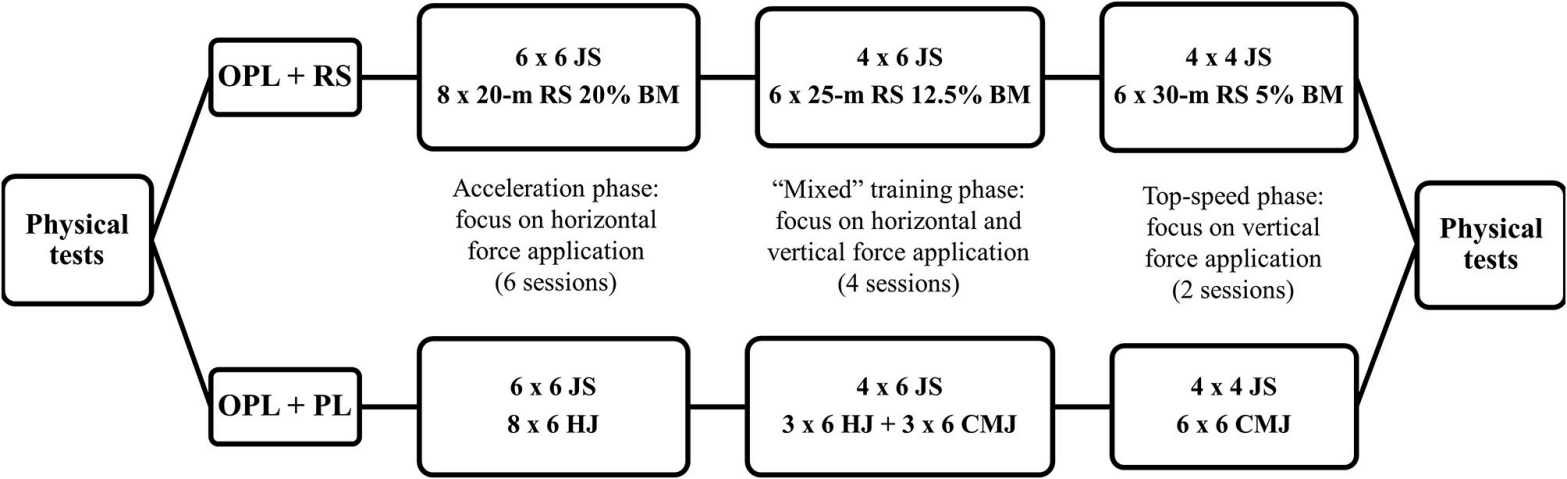
Training content across the 5-week preseason period for both groups of training. OPL: optimum power load; RS: resisted sprint; PL: vertical and horizontal plyometrics; JS: jump squat; HJ: horizontal jump; CMJ: countermovement jump; BM: body-mass.

In this study, a parallel two-group, randomized, longitudinal design was conducted to test the effectiveness of two mixed power-oriented training programs on the neuromuscular performance of elite soccer players. All athletes had been previously familiarized with the performance tests. Unloaded and resisted sprinting speeds at 5-, 10-, 20-, and 30-m, change of direction (COD) speed, maximum mean propulsive power (MPP) relative to the athletes' body mass in the jump squat exercise (JS), mean propulsive velocity (MPV) using 40% of the players' BM in the JS, and performance in the squat jump (SJ), countermovement jump (CMJ), and horizontal jump (HJ) were assessed pre- and post-training period. For the “OPL + RS” group, MPP JS and resisted sprint velocity were the independent variables, whereas vertical and horizontal jump distances, unloaded sprint velocities, COD speed, and MPV were the dependent variables. For the “OPL + PL” group, MPP JS and vertical and horizontal jump distances were the independent variables, whereas resisted and unloaded sprint velocities, COD speed, and MPV were the dependent variables. During the resisted sprints, athletes carried a sled towing device attached to a waistcoat as described elsewhere (Cronin and Hansen, 2006). Prior to all testing sessions, a general and specific warm-up routine was performed, involving light running (5-min at a self-selected pace) and submaximal attempts at each testing exercise (e.g., submaximal sprints and vertical jumps). During the experimental period, all soccer players performed 12 power-oriented training sessions. A typical weekly training schedule, as well as the detailed power-oriented training program across the 5-week preseason period, are presented in Table 1 and Figure 1, respectively.

**Table 1:** Typical weekly training program for the soccer players during the 5 weeks of intervention.

	**Monday**	**Tuesday**	**Wednesday**	**Thursday**	**Friday**
**Morning**	TEC/TAC 60′	ST/PT 30′	Rest	ST/PT 30′	Rest
**Afternoon**	TEC/TAC 60′	TEC/TAC 80′	TEC/TAC 90′	TEC/TAC 80′	TEC/TAC 90′

*TEC = Technical Training; TAC = Tactical Training; ST/PT = Strength-Power Training (optimum power load + resisted sprint or optimum power load + vertical and horizontal plyometrics); In the third and fourth weeks 3 ST/PT training sessions were performed. TEC/TAC training involved different formats of small-sided games*.

### Vertical jumping tests

Vertical jumping height was determined using both SJ and CMJ. In the SJ, subjects were required to remain in a static position with a 90° knee flexion angle for 2-s before jumping. In the CMJ, the soccer players were instructed to execute a downward movement followed by a complete extension of the legs. The SJ and CMJ were executed with the hands fixed on the hips. All jumps were performed on a contact platform (Elite Jump System®; S2 Sports, São Paulo, Brazil) (Loturco et al., 2017). A total of five attempts were allowed for each jump, interspersed by 15-s. The best attempts at SJ and CMJ were retained.

### Horizontal jumping assessment

Athletes performed the HJ starting from a standing position. They commenced the jump by swinging their arms and bending their knees to provide maximal forward drive. The jump-length measurement was determined using a metric tape measure (Lufkin, L716MAGCME, Appex Group, USA), from the take-off line to the nearest point of landing contact (i.e., back of the heels). Each athlete executed three attempts, interspersed by 30-s intervals, and the longest jump was retained for the analyses.

### Bar mean propulsive power in jump squat

Maximum MPP in the JS exercise was assessed on a Smith machine (Hammer Strength, Rosemont, IL, USA). Players were instructed to execute two repetitions at maximal velocity for each load, starting at 40% of their BM. Athletes executed a knee flexion until the thigh was parallel to the ground (≈100° knee angle) and, after a command, jumped as fast as possible without losing contact between their shoulder and the bar. A load of 10% BM was gradually added until a decrease in mean propulsive power was observed. A 5-min interval between sets was provided. To determine MPP, a linear transducer (T-Force, Dynamic Measurement System; Ergotech Consulting S.L., Murcia, Spain) was attached to the Smith machine bar. The technical specification of the MPP analysis and its calculation have been described previously (Loturco et al., 2017a). The load corresponding to the maximum MPP value was considered as the “OPL” and used in the muscle power-oriented training sessions for both groups. Due to its established relationships with speed and power performances (Loturco et al., 2014, 2015a), the MPV associated to 40% of the players' BM (MPV 40%) was retained. In addition, the maximum MPP value normalized by the players' BM [i.e., relative values (MPP REL)] was considered for data analysis.

### Sprinting speed

Five pairs of photocells (Smart Speed, Fusion Equipment, AUS) were positioned at distances of 0, 5-, 10-, 20-, and 30-m along the sprinting course. The soccer players sprinted four times (2 RS with a load of 20% of the players' BM and 2 unloaded sprints), starting from a standing position 0.3 m behind the starting line. To avoid weather influences, the sprint tests were performed on an indoor running track. A 5-min rest interval was allowed between the two attempts and the fastest time for both types was considered for the analyses.

### Zig-zag change of direction speed test

**Figure 2:**
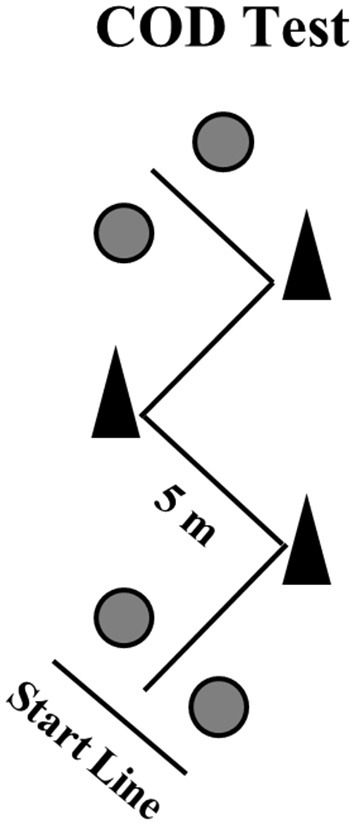
A schematic presentation of the change of direction (COD) speed test. The gray circles represent the position of the photocells.

The COD course consisted of four 5-m sections marked with cones set at 100° angles, on an indoor court (Figure 2) (Little and Williams, 2005). The athletes were required to decelerate and accelerate as fast as possible without losing body stability. Two maximal attempts were performed with a 5-min rest interval between attempts. Starting from a standing position with the front foot placed 0.3-m behind the first pair of photocells (i.e., starting line), the athletes ran and changed direction as quickly as possible, until crossing the second pair of photocells, placed 20-m from the starting line. The fastest time from the two attempts was retained for analyses.

### Statistical analysis

Data are presented as mean ± standard deviation (SD). To analyze the differences in the vertical jumps, sprinting times, COD speed, vertical and horizontal jumps, and MPV 40% in the OPL + RS and OPL + PL, pre- and post-training, the differences based on magnitudes were calculated (Batterham and Hopkins, 2006). The quantitative chances of the OPL + RS and OPL having higher, similar or lower values were assessed qualitatively as follows: <1%, almost certainly not; 1 to 5%, very unlikely; 5 to 25%, unlikely; 25 to 75%, possible; 75 to 95%, likely; 95 to 99%, very likely; >99%, almost certain. If the chances of having better and poorer results were both >5%, the true difference was assessed as unclear. Additionally, to determine the magnitude of the differences between the groups pre- and post-training and its delta changes, the standardized differences (ES: Cohen's *d*) were calculated (Hopkins et al., 2009). The ES magnitudes were interpreted using the following thresholds: <0.2, 0.2–0.6, 0.6–1.2, 1.2–2.0, 2.0–4.0, and >4.0 for trivial, small, moderate, large, very large, and near perfect, respectively (Hopkins et al., 2009). All performance tests used in this research presented good levels of absolute and relative reliability (CV < 5% and ICC > 0.90 for all assessments) (Hopkins et al., 2009).

## Results

All data presented normal distribution. On average, soccer players of both training groups achieved their maximum MPP values with a load corresponding to 60.1 ± 2.3% of their BM. Table 2 shows the comparisons of the vertical and horizontal jumps, MPV 40%, MPP REL, and COD speed pre- and post- the preseason period for both groups of power-oriented training.

**Table 2:** Comparisons of the squat jump (SJ), countermovement jump (CMJ), and horizontal jump (HJ), mean propulsive velocity (MPV) using a load of 40% of the players' body mass in the jump squat exercise, and change of direction (COD) speed pre- and post- a 5-week preseason period for the groups of elite soccer players that performed two different power-oriented training regimes.

	**Optimum power load** + **resisted sprints**			**Optimum power load** + **plyometrics**		
	**Pre**	**Post**	**ES (90% CL) *rating***	**Pre**	**Post**	**ES (90% CL) *rating***
SJ (cm)	40.6 ± 2.8	40.3 ± 2.7	−0.09 (−0.79; 0.60) *trivial*	38.7 ± 3.4	39.6 ± 3.0^P^	0.25 (−0.07; 0.57) *small*
CMJ (cm)	41.8 ± 2.9	40.6 ± 3.7	−0.39 (−1.03; 0.25) *small*	39.0 ± 3.9	39.8 ± 3.3^P^	0.20 (−0.15; 0.55) *small*
HJ (m)	2.46 ± 0.22	2.45 ± 0.21	−0.03 (−0.37; 0.30) *trivial*	2.28 ± 0.20	2.37 ± 0.15^L^	0.40 (0.15; 0.66) *small*
MPV 40% (m.s^−1^)	1.27 ± 0.10	1.23 ± 0.11	−0.34 (−1.04; 0.35) *small*	1.29 ± 0.09	1.30 ± 0.06	0.08 (−0.39; 0.55) *trivial*
MPP REL JS (W.kg^−1^)	9.54 ± 0.87	9.41 ± 0.96	−0.13 (−0.64; 0.38) *trivial*	9.75 ± 0.85	9.69 ± 0.77	−0.06 (−0.46; 0.33) *trivial*
COD (s)	5.93 ± 0.15	5.78 ± 0.19^L^	−0.86 (−1.55; −0.17) *moderate*	6.10 ± 0.20	5.91 ± 0.25^AC^	−0.87 (−1.18; −0.56) *moderate*

ES: standardized differences based on Cohen units; CL: confidence limits;

P possibly different from pre;

L likely different from pre;

AC *almost certainly different from pre*.

**Figure 3:**
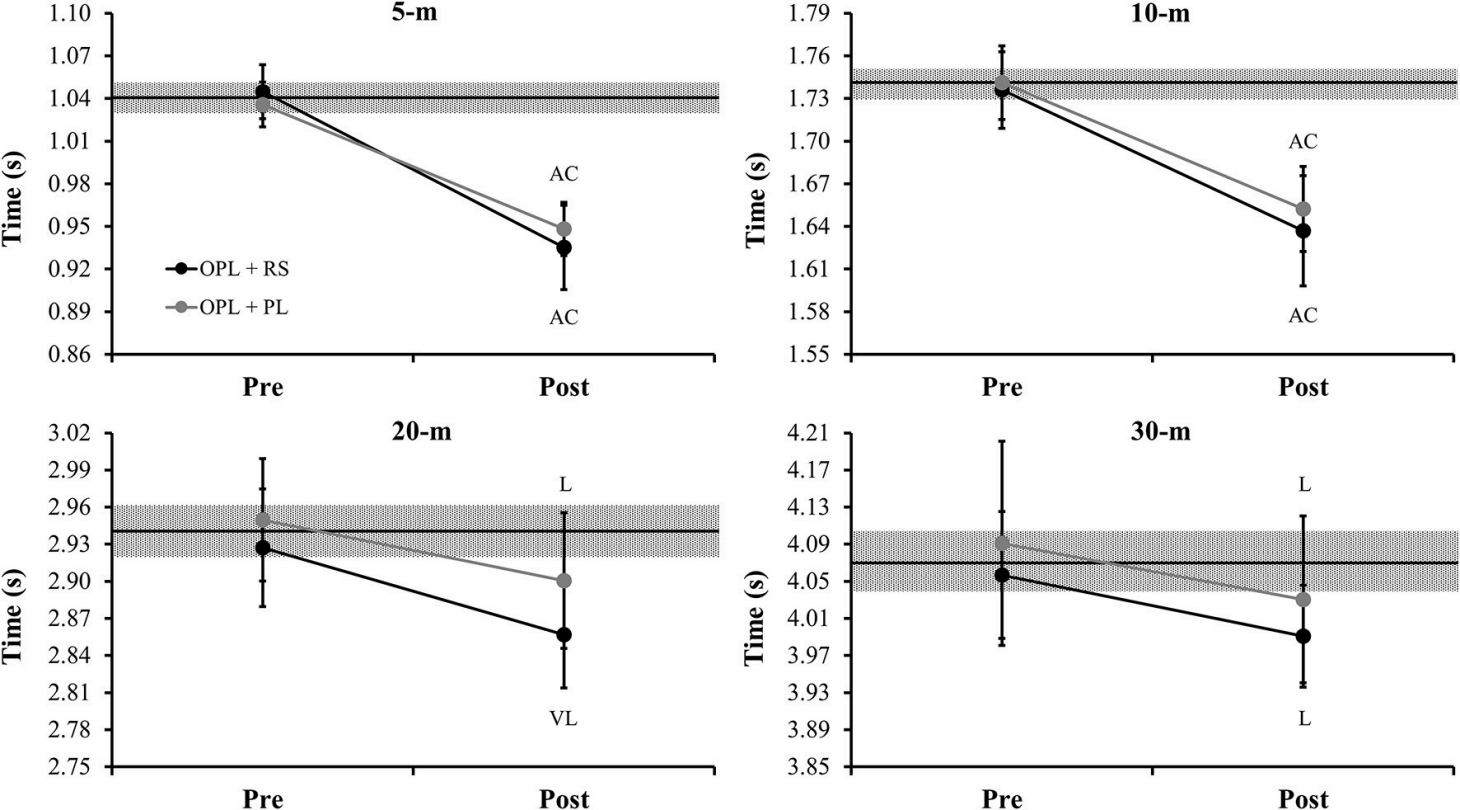
Comparisons of the unloaded sprint times in 5-, 10-, 20-, and 30-m pre- and post- the preseason period for both power-oriented training groups. OPL: optimum power load; RS: resisted sprint; PL: vertical and horizontal plyometrics; middle black lines represent the mean values between two groups in the “pre” measures; gray areas represent the smallest worthwhile change (0.2 × between subject SD); Error bars represent 90% confidence limits; AC: almost certainly difference in comparison to pre-values; VL: very likely difference in comparison to pre-values; L: likely difference in comparison to pre-values.

Figure 3 depicts the comparisons of the unloaded sprint times in 5-, 10-, 20-, and 30-m pre- and post- the preseason period for both power-oriented training groups. Both training groups demonstrated a *likely* to *almost certainly* decrease in the sprint times comparing pre- and post-assessments.

**Figure 4:**
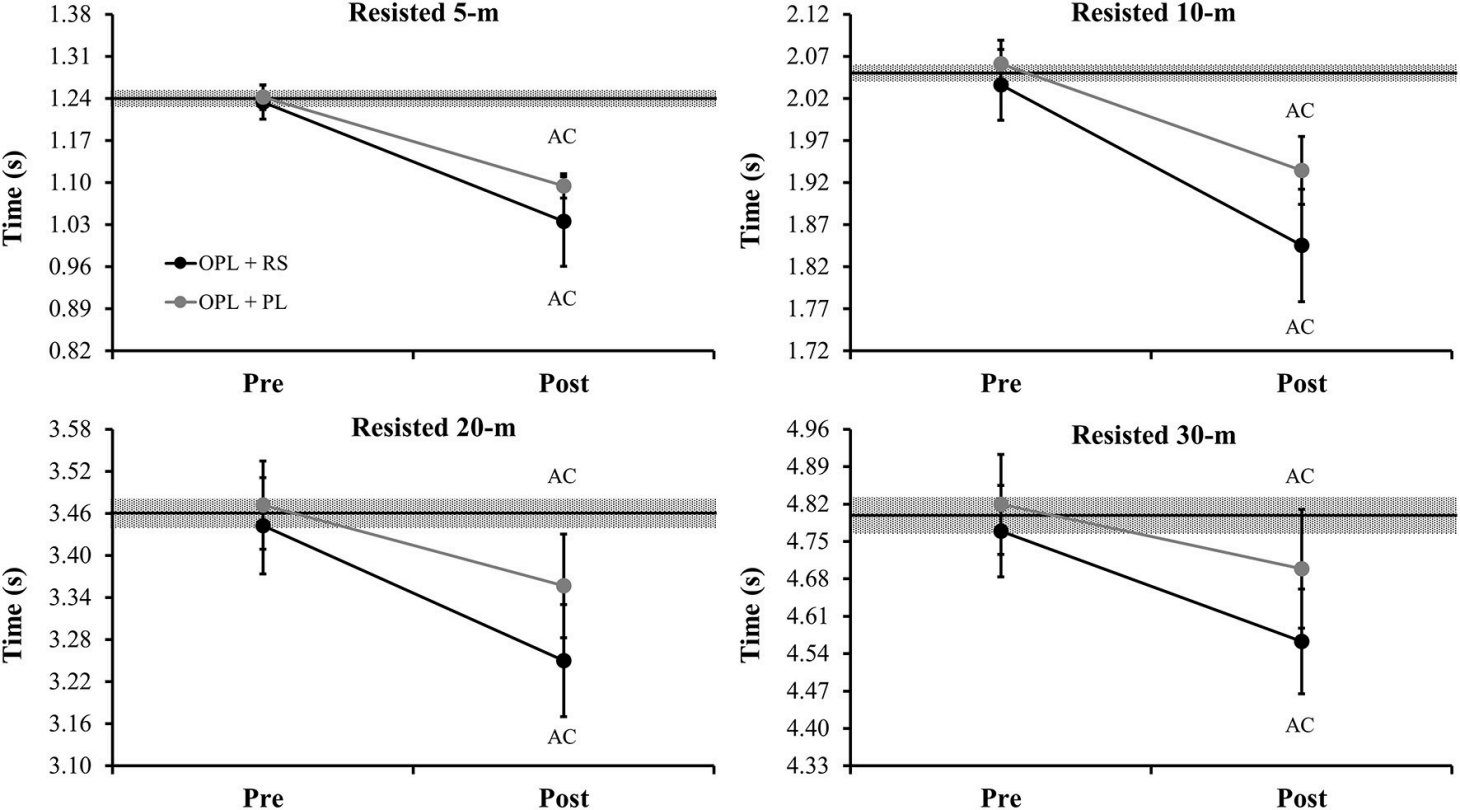
Comparisons of the resisted sprint times in 5-, 10-, 20-, and 30-m, using a load of 20% of the players' body-mass, pre- and post- the preseason period for both power-oriented training groups. OPL: optimum power load; RS: resisted sprint; PL: vertical and horizontal plyometrics; middle black lines represent the mean values between two groups in the pre-measures; gray areas represent the smallest worthwhile change (0.2 × between subject SD); Error bars represent 90% confidence limits; AC: almost certainly difference in comparison to pre-values.

**Figure 5:**
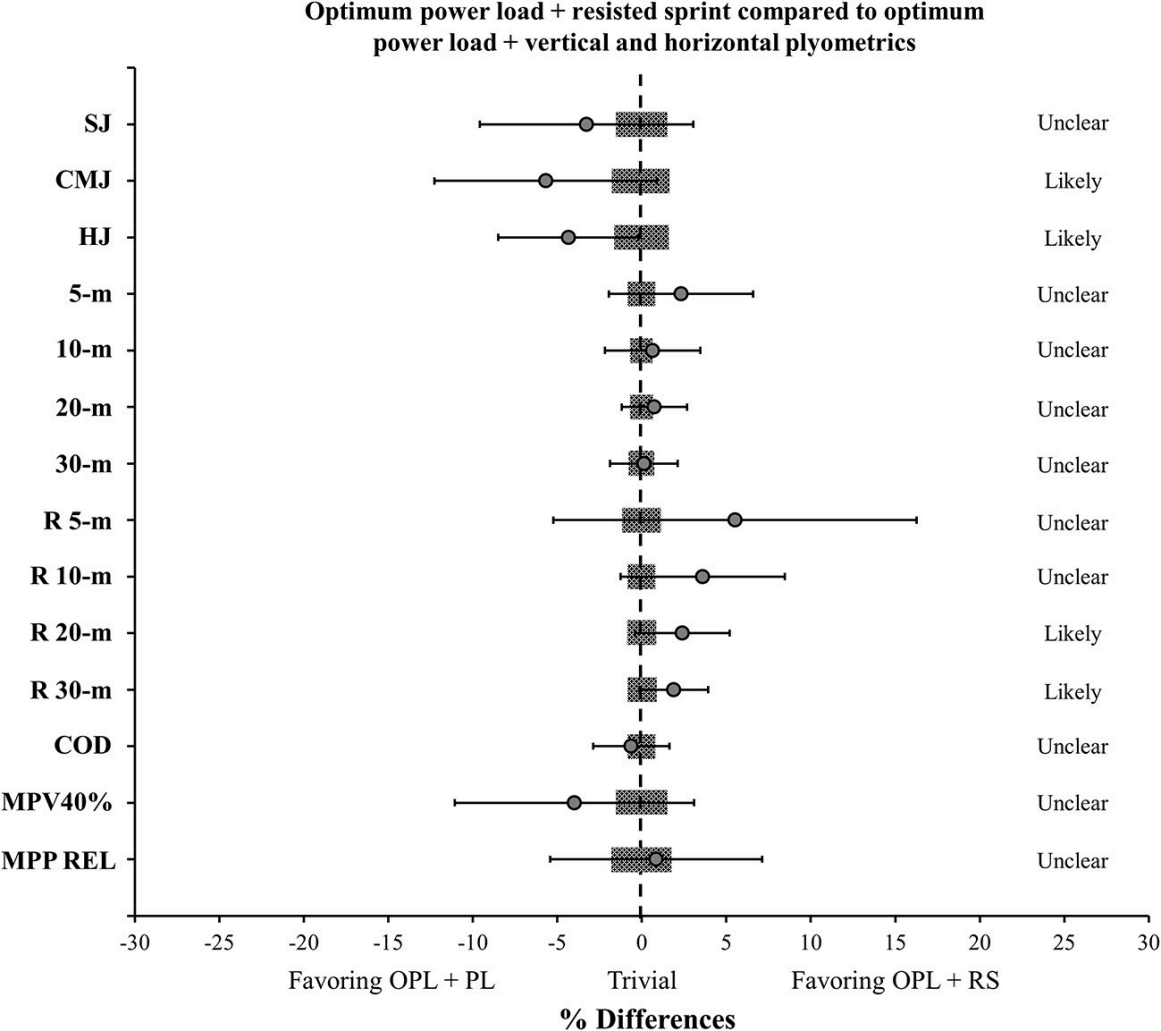
Comparisons of the delta changes in the variables tested between the two power-oriented training regimes. Gray areas represent the smallest worthwhile change (0.2 × between subject SD) for each variable; OPL: optimum power load; RS: resisted sprint; PL: vertical and horizontal plyometrics; error bars represent 90% confidence limits. SJ: squat jump; CMJ: countermovement jump; HJ: horizontal jump; R: resisted sprints with a load of 20% of the players' body-mass; COD: change of direction speed test; MPV 40%: mean propulsive velocity in the jump squat exercise with a load of 40% of the players' body-mass; MPP REL: mean propulsive power in the jump squat exercise relative to the players' body-mass.

Figure 4 depicts the comparisons of the resisted sprint times in 5-, 10-, 20-, and 30-m, using a load of 20% of the players' BM, pre- and post- the preseason period for both power-oriented training groups. Both training groups demonstrated an *almost certainly* decrease in the resisted sprint times at the end of the preseason training period. Finally, Figure 5 describes the comparisons of the delta changes in all variables tested between the two power-oriented training regimes (OPL + RS vs. OPL + PL).

## Discussion

To our knowledge, this is the first study to compare the effects of two mixed training strategies, which combined OPL and different types of speed-specific exercises, on neuromechanical capacities of elite soccer players. From a general perspective, both training modes were effective in improving the speed-related performance indices during the short preseason. In contrast, vertical and horizontal jumping abilities increased meaningfully only in the athletes exposed to plyometric exercises, showing to be highly dependent on training specificity. Lastly, in both groups, the mechanical variables related to muscle power development (i.e., MPV 40% BM and MPP REL JS) did not change after the investigated training period.

The absence of worthwhile improvements in muscle power capacity seems to be commonplace in investigations performed throughout soccer preseasons (Faude et al., 2013; Loturco et al., 2015d). Importantly, it appears that this phenomenon is independent of training strategy, age-category or player's background (i.e., previous experience and training status) (Los Arcos et al., 2014). It is probable that the high volume of predominantly aerobic workouts (technical and tactical sessions) typically performed during these phases precludes the proper development of muscle power, due to the well-documented concurrent training effects between strength and endurance adaptations (Fyfe et al., 2014; Loturco et al., 2015d). Thus, when power capacity is the main objective of the training period, it is highly recommended that coaches consider the possibility of reducing the relative content of soccer-specific activities, while at the same time increasing the volume (and frequency) of neuromuscular training (Bird et al., 2005; Fyfe et al., 2014). For example, this “load-balancing strategy” has been shown to be quite effective in enhancing strength-power qualities of professional players during the inter-season intermission between two consecutive elite soccer championships (Loturco et al., 2016a), where the aerobic loading is naturally reduced in a progressive and gradual manner (when compared to the preseason phases) (Jeong et al., 2011).

Vertical and horizontal jump distances are regularly used as valid and reliable measures of leg explosiveness in team sport athletes (Maulder and Cronin, 2005; Loturco et al., 2015c,e). Besides this, the considerable number of jumps executed during training and official matches makes it essential to asses and develop this capacity in professional soccer player (Liu et al., 2016). That said, it is important to note that the jump performance increased only in the “plyometric group” (Table 2), confirming the theoretical conclusions of previous research which has already defined that top-level athletes may not record improvements in this ability without executing specific jump exercises (Baker, 1996). These positive results have also been observed in numerous studies involving elite soccer players, who similarly reported significant enhancements in unloaded jump performance after executing isolated or combined (e.g., complex training) plyometric training programs (Maio Alves et al., 2010; Loturco et al., 2015e). For many authors, the specificity of these neuromechanical responses reinforces the importance of combining traditional lifts with movements that make use of the stretch-shortening cycle, mainly when there is a clear intention to maximize the jumping potential (Baker, 1996; Maio Alves et al., 2010). Therefore, coaches and sport scientists interested in improving the unloaded jump capacities of professional soccer players are strongly encouraged to systematically include plyometric exercises in their strength-power training routines (Loturco et al., 2016b).

The most important finding reported here is that, independent of the training intervention, both groups reported substantial increases in maximal sprint and COD performances. Notably, the speed improvements occurred over a wide range of distances (from 5- to 30-m), without meaningful differences between-groups. To some extent, these outcomes may be explained by analyzing more closely the experimental training designs. Whereas the plyometric group combined vertical and horizontal jumps, the resisted sprint group used workloads ranging from 5 to 20% of BM. From a mechanical perspective, both horizontally oriented exercises and heavy sled resisted sprint tend to produce greater increases in the initial phase of sprinting, which is possibly related to the critical importance of horizontal force production during this phase (Bachero-Mena and Gonzalez-Badillo, 2014; Loturco et al., 2015e; Petrakos et al., 2016). Indeed, previous investigations have shown that horizontal plyometrics and resisted sprints with heavier loads (i.e., 20% BM) should be preferred to improve short-distance acceleration performance (up to10-m) (Bachero-Mena and Gonzalez-Badillo, 2014; Petrakos et al., 2016). In contrast, vertical jumps and resisted sprints with lighter loads (i.e., from 5 to 12.5% BM) might be more effective to maximize speed at longer distances (i.e., “top-speed phases”), usually characterized by shorter contact times with associated increases in vertical peak forces (also related to increases in vertical jump height) (Weyand et al., 2000; Bachero-Mena and Gonzalez-Badillo, 2014; Loturco et al., 2015c; Petrakos et al., 2016). Correspondingly, a recent systematic review suggests that sprint adaptations could be velocity specific-dependent, with heavier sled loads (> 20% BM) improving initial acceleration (characterized by slow velocities and high resistive forces) and light sled loads (<10%BM) improving the maximal velocity phase (characterized by high velocity and low resistive forces) (Petrakos et al., 2016). This “multifaceted nature” of both training programs could also have contributed to the worthwhile increases found in COD performance, widely recognized as a complex and heterogenous physical ability (Sheppard and Young, 2006). From these results, it is suggested that the combination of exercises and workloads able to induce positive responses over different phases of sprint running may be a very useful strategy in top-level sports. This is particularly true in modern soccer, where professional players are required to progressively develop their speed-related abilities to cope with the demands related to their involvement in high-intensity match (and training) activities (Barnes et al., 2014; Haugen et al., 2014).

It is worth emphasizing that maximal sprint running depends directly on relative mechanical power production (i.e., W. Kg^−1^) (Morin et al., 2012). Yet, in this context, many studies involving elite athletes have already reported strong positive correlations between these variables (Cronin and Hansen, 2005; Loturco et al., 2015c, 2017a). However, interestingly, the meaningful improvements in speed reported above, occurred regardless of the absence of increases in muscle power capacity. Based on these results, it may be inferred that both training schemes could lead to better and more effective application of the resultant force vector onto the ground while sprinting, which has been previously described as a key determinant of sprint performance (Morin et al., 2012). As such, according to the training designs (Figure 1), it is possible that our subjects initially improved their ability to apply force with a forward orientation over the distinct acceleration phases and (Morin et al., 2012), latterly, their ability to apply force with a vertical orientation at higher speeds (Weyand et al., 2000; Loturco et al., 2015c). This holds true even for the resisted sprint group, since the very light loads (5% BM) adopted in the “top-speed phase” allowed soccer players to sprint near to their maximal velocities, which are directly related to relative average vertical force production (Weyand et al., 2000). Although the mechanisms underlying these phenomena are not fully understood, the prevalent view is that the force vector theory may play a crucial role in modulating the chronic training responses (Randell et al., 2010; Contreras et al., 2017). Therefore, this “enhanced capability” to orient the force vector while sprinting seems to be a positive and important technical change provided by both mixed training modes used herein, and it is independent of the possible adaptations in muscle power capacity.

As with many investigations performed with top-level athletes during their professional training routines, this study is limited by several factors, such as the absence of a control group, the small sample size (i.e., an elite soccer team), and the impossibility of controlling the total training content. Nevertheless, it is critical to emphasize that both mixed training modes used herein were effective in increasing the speed-related performance of professional soccer players during a short preseason, which has been considered a great challenge (and an obvious goal) for coaches and sport scientists (Barnes et al., 2014; Loturco et al., 2015d).

In summary, this study shows that a mixed training approach which comprises exercises and workloads able to produce positive adaptations in different phases of sprinting can be a very effective strategy in team-sports, especially in professional soccer, where maximal acceleration and speed capacities play determinant roles in optimizing performance (Barnes et al., 2014; Haugen et al., 2014). Moreover, the possibility of combining optimum power loads with these complementary workouts (i.e., resisted sprints or plyometrics) emerges as a novel and suitable option for coaches and sport scientists, due to the applicability, efficiency and time-saving characteristics of this strength-power training approach (Loturco et al., 2016a).

## Conclusion

Coaches and sport scientists are constantly seeking practical and efficient alternatives to improve maximal running speed and acceleration capacities in team-sport athletes. This is especially true in modern soccer, where high-intensity activities can greatly influence the match outcome (Barnes et al., 2014; Haugen et al., 2014; Loturco et al., 2015e). Our data provide valuable information regarding the gradual development of sprint performance in soccer athletes, even during short pre-seasons, typically characterized by high volumes of concurrent endurance training (i.e., specific technical-tactical sessions) (Faude et al., 2013; Loturco et al., 2015d). Based on the findings reported here, strength and conditioning specialists are strongly encouraged to combine in the same training program workouts targeted at increasing speed qualities more related to initial phases of sprinting (i.e., heavy sled loads and horizontal jumps) or to “top-speed” phases (i.e., light sled loads or vertical plyometrics). Simultaneously, to reduce the interference effect of aerobic loads and maintain the muscle power ability during the preseason period, a strength-power training regimen based on optimum power loads could be implemented. As previously reported, this is a very effective and applied training strategy, which is an essential aspect in professional soccer, due to its congested training, traveling and playing schedules (Loturco et al., 2016a,c). Further studies should be designed to assess and compare the effectiveness of more mixed training combinations (e.g., plyometrics + resisted sprints + different strength-power exercises) in improving the plethora of all field-related physical capacities (e.g. jump, speed, and power abilities) demanded by professional soccer and other elite team-sports.

## Author contributions

Designed the work: IL; data acquisition: IL, RK, KK, CC, BF, LA, LP; analysis and interpretation of data: IL, LP; drafting the work: IL, LP; revising critically the work: IL, RK, KK, CC, BF, LA, LP; final approval of the version to be published: IL, RK, KK, CC, BF, LA, LP; agree to be accountable for all aspects of the work in ensuring that questions related to the accuracy or integrity of any part of the work were appropriately investigated and resolved: IL, RK, KK, CC, BF, LA, LP.

### Conflict of interest statement

The authors declare that the research was conducted in the absence of any commercial or financial relationships that could be construed as a potential conflict of interest.
